# Supplementary material for: Heat adaptation of phage T7 under an extended genetic code
Source: Virus Evol. 2021 Dec 1;7(2):veab100. doi: 10.1093/ve/veab100 (PMC8923235; doi:10.1093/ve/veab100)
Supplement: veab100_Supp [file veab100_supp.zip › Sequencing_Analysis.zip!/Sequencing_Analysis/S3/evidence/JC_53.html]

BRESEQ :: Evidence


| New junction evidence | | | | | | | | | | | |
| --- | --- | --- | --- | --- | --- | --- | --- | --- | --- | --- | --- |
|  | | seq id | position | reads (cov) | reads (cov) | score | skew | freq | annotation | gene | product |
| \* | ? | NC\_001604.1 | = 16604 | 551 (0.320) | 655 (0.390) | 233/590 | 1.5 | 51.6% | intergenic (–/–) | *–/–* | –/– |
|  | ? | NC\_001604.1 | 17028 = | 688 (0.410) |  |  |  |  | intergenic (–/–) | *–/–* | –/– |

Only 100 of 741 total aligned reads displayed.  

`GCATGGGTACATGATGAAATCCAAGTAGGCTGCCGTACCGAAGAGATTGCTCAGGTGGTCATTGAGACCGCACAAGAAGCGATGCGCTGGGTTGGAGACCACTGGAACTTCCGGTGTCTTCTGGATACCGAAGGTAAGATGGGTCCTAATTGGGCGATTTGCCACTGATACAGGAGGCTACTCATGAACGAAAGACACTTAACAGGTGCTGCTTCTGAAATGCTAGTAGCCTACAAATTTACCAAAGCTGGGTACACTGTCTATTACCCTATGCTGACTCAGAGTAAAGAGGACTTGGTTGTATG‑‑‑‑‑‑‑‑‑‑‑‑‑‑‑‑‑‑‑‑‑‑‑‑‑‑‑‑‑‑‑‑‑‑‑‑‑‑‑‑‑‑‑‑‑‑‑‑‑‑‑‑‑‑‑‑‑‑‑‑‑‑‑‑‑‑‑‑‑‑‑‑‑‑‑‑‑‑‑‑‑‑‑‑‑‑‑‑‑‑‑‑‑‑‑‑‑‑‑‑‑‑‑‑‑‑‑‑‑‑‑‑‑‑‑‑‑‑‑‑‑‑‑‑‑‑‑‑‑‑‑‑‑‑‑‑‑‑‑‑‑‑‑‑‑‑‑‑‑‑‑‑‑‑‑‑‑‑‑‑‑‑‑‑‑‑‑‑‑‑‑‑‑‑‑‑‑‑‑‑‑‑‑‑‑‑‑‑‑‑‑‑‑‑‑‑‑‑‑‑‑‑‑‑‑‑‑‑‑‑‑‑‑‑‑‑‑‑‑‑‑‑‑‑‑‑‑‑‑‑‑‑‑‑‑‑‑‑‑‑‑‑‑‑‑‑‑‑‑‑‑‑‑‑‑‑‑‑‑‑‑‑‑‑‑‑‑‑‑‑‑‑‑‑‑‑‑‑‑‑‑‑‑‑‑‑‑‑‑‑‑‑‑‑‑‑‑‑  >  NC_001604.1/16300‑16604`  
`‑‑‑‑‑‑‑‑‑‑‑‑‑‑‑‑‑‑‑‑‑‑‑‑‑‑‑‑‑‑‑‑‑‑‑‑‑‑‑‑‑‑‑‑‑‑‑‑‑‑‑‑‑‑‑‑‑‑‑‑‑‑‑‑‑‑‑‑‑‑‑‑‑‑‑‑‑‑‑‑‑‑‑‑‑‑‑‑‑‑‑‑‑‑‑‑‑‑‑‑‑‑‑‑‑‑‑‑‑‑‑‑‑‑‑‑‑‑‑‑‑‑‑‑‑‑‑‑‑‑‑‑‑‑‑‑‑‑‑‑‑‑‑‑‑‑‑‑‑‑‑‑‑‑‑‑‑‑‑‑‑‑‑‑‑‑‑‑‑‑‑‑‑‑‑‑‑‑‑‑‑‑‑‑‑‑‑‑‑‑‑‑‑‑‑‑‑‑‑‑‑‑‑‑‑‑‑‑‑‑‑‑‑‑‑‑‑‑‑‑‑‑‑‑‑‑‑‑‑‑‑‑‑‑‑‑‑‑‑‑‑‑‑‑‑‑‑‑‑‑‑‑‑‑‑‑‑‑‑‑‑‑‑‑‑‑‑‑‑‑‑‑‑‑‑‑‑‑‑‑‑‑‑‑‑‑‑‑‑‑‑‑‑‑‑‑‑‑‑‑gtatgGAAGGATTGATGACATTCGTAGTACGTACATCATTTCGTGAGGCCATTAAGGACATGCACGAAGAGTATGCAGATAAGGACTCTTTCAAACAATCTCCTGCAACAGTACGGGAGGTGTTCTGATGTCTGACTACCTGAAAGTGCTGCAAGCAATCAAAAGTTGCCCTAAGACTTTCCAGTCCAACTATGTACGGAACAATGCGAGCCTCGTAGCGGAGGCCGCTTCCCGTGGTCACATCTCGTGCCTGACTACTAGTGGACGTAACGGTGGCGCTTGGGAAATCACTGCTTCCGGTAC  >  NC_001604.1/17028‑17325`  

                                                                                                                                                                                                                                                                                                                                                                                                                                                                                                                                                                                                                             
`GCATGGGTACATGATGAAATCCAAGTAGGCTGCCGTACCGAAGAGATTGCTCAGGTGGTCATTGAGACCGCACAAGAAGCGATGCGCTGGGTTGGAGACCACTGGAACTTCCGGTGTCTTCTGGATACCGAAGGTAAGATGGGTCCTAATTGGGCGATTTGCCACTGATACAGGAGGCTACTCATGAACGAAAGACACTTAACAGGTGCTGCTTCTGAAATGCTAGTAGCCTACAAATTTACCAAAGCTGGGTACACTGTCTATTACCCTATGCTGACTCAGAGTAAAGAGGACTTGGTTG                                                                                                                                                                                                                                                                                                                <  1:197451/301‑1`  
   `ggaataagagacagAATCCAAGTAGGCTGCCGTACCGAAGAGATTGCTCAGGTGGTCATGGAGACCGCACAAGAAGCGATGCGCTTGGTTGGAGACCACTGGAACTTCCGGTGGCTTCTGGAAACCGAAGTTAAGATGGGTCCTAATTGGGCGAATTGCCACTGATACAGGAGGCTACTCATGGACGAAAGACACTTAACAGGTGCTGGTTCTGAAATGCTAGTATCCTACAAATTGACCAAAGCTGGGTACACTGTCTATTACACTATGCTGACTCAGAGTAAAGAGGACTTGGTTGTAT                                                                                                                                                                                                                                                                                                             <  1:340394/287‑1`  
   `TGGGTACATGATGAAATCCAAGTAGGCTGCCGTACCGAAGAGATTGCTCAGGTGGTCATTGAGACCGCACAAGAAGCGATGCGCTGGGTTGGAGACCACTGGAACTTCCGGTGTCTTCTGGATACCGAAGGTAAGATGGGTCCTAATTGGGCGATTTGCCACTGATACAGGAGGCTACTCATGAACGAAAGACACTTAACAGGTGCTGCTTCTGAAATGCTATTAGCCTACAAATTTACCAAAGCTGGGTACACTGTCTATTACCCTATTCTGACTCAGAGTAAAGAGGACTTGGTTGTAT                                                                                                                                                                                                                                                                                                             >  1:166156/1‑301`  
    `GGGTACATGATGAAATCCAAGTAGGCTGCCGTACCGAAGAGATTGCTCAGGTGGTCATTGAGACCGCACAAGAAGCGATGCGCTGGGTTGGAGACCACTGGAACTTCCGGTGTCTTCTGGATACCGAAGGTAAGATGGGTCCTAATTGGGCGATTTGCCACTGATACAGGAGGCTACTCATGAACGAAAGACACTTAACAGGTGCTGCTTCTGAAATGCTAGTAGCCTACAAATTTACCAAAGCTGGGTACACTGTCTATTACCCTATGCTGACTCAGAGTAAAGAGGACTTGGTTGTATG                                                                                                                                                                                                                                                                                                            >  1:269760/1‑301`  
     `gaTACATGATGAAAACCAAGTAGGCTGCCGTACCGAAAAGATTGCTCAAGTTGTCATTGAGACCGCACAGGAAGCGATACGCTGGGTTGGAGACCAATGGAACTTCCGGTGGCTTCTGGATACCGGAGGTAAGATGGGTCCTAATTGGGAGATTTGCCACTGATACAGGAGGCTACTCATGAACAAAAGACACTTAACAGGTGCTGCTTTTGAAATGCTAGTAGCCTACAAATTTACCGAAACTGGGTACACTGTCTATTACCCTATGCTGACTCAGAGTAAAGAGGACTTGGTTGTATGG                                                                                                                                                                                                                                                                                                           <  1:307703/299‑1`  
        `gtataagagacagCAAGTAGGCTGCCGTACCGAAGAGATTGCTCAGGTGGTCATTGAGACCGCACAAGAAGCGATGCGCTGGGTTGGAGACCACTGGAACTTCCGGTGTCTTCTGGATACCGAAGGTAAGATGGGTCCTAATTGGGCGATTTGCCACTGATACAGGAGGCTACTCATGAACGAAAGACACTTAACAGGTGCTGCTTCTGAAATGCTAGTAGCCTACAAATTTACCAAAGCTGGGTACACTGTCTATTACCCTATGCTGACTCAGAGTAAAGAGGACTTGGTTGTATGGAAG                                                                                                                                                                                                                                                                                                        <  1:225844/288‑1`  
             `ATGAAATCCAAGTAGGCTGCCGTACCGAAGAGATTGCTCAGGTGGTCATTGAGACCGCACAAGAAGCGATGCGCTGGGTTGGAGACCACTGGAACTTCCGGTGTCTTCTGGATACCGAAGGTAAGATGGGTCCTAATTGGGCGATTTGCCACTGATACAGGAGGCTACTCATGAACGAAAGACACTTAACAGGTGCTGCTTCTGAAATGCTAGTAGCCTACAAATTTACCAAAGCTGGGTACACTGTCTATTACCCTATGCTGACTCAGAGTAAAGAGGACTTGGTTGTCTGGAAGGATTG                                                                                                                                                                                                                                                                                                   >  1:343607/1‑301`  
             `ATGAAATCCAAGTAGGCTGCCGTACCGAAGAGATTGCTCAGGTGGTCATTGAGACCGCACAAGAAGCGATGCGCTGGGTTGGAGACCACTGGAACTTCCGGTGTCTTCTGGATACCGAAGGTAAGATGGGTCCTAATTGGGCGATTTGCCACTGATACAGGAGGCTACTCATGAACGAAAGACACTTAACAGGTGCTGCTTCTGAAATGCTAGTAGCCTACAAATTTACCAAAGCTGGGTACACTGTCTATTACCCTATGCTGACTCAGAGTAAAGAGGACTTGGTTGTATGGAAGGATTG                                                                                                                                                                                                                                                                                                   <  1:123110/301‑1`  
                `AAAGCCAAGTAGGCTGACGTACAGAAGAGAATGATCAGGTGGTCATTGAGACCGAACAAGAAGCGATGCGCTGGGTTGGAGACCACTGGAACTTCCGGTGTCTTCTGGATACCGAAGGTAAGATGGGTCCTAATTGGGAGATTTGCCACTGATACAGGAGGCTACTCATGAACGAAAGACACTTAACAGGTGCTGCTTCTGAAATGCTAGTAGCCTACAAATTTACCAAAGCTGGGTACACTGTCTATTACCCTATGCTGACTCAGAGTAAAGAGGACTTGGTTGTATGGAAGGATTGATG                                                                                                                                                                                                                                                                                                <  1:219501/301‑1`  
                 `AATCCAAGTAGGCTGCCGTACCGAAGAGATTGCTCAGGTGGTCATTGAGACCGCACAAGAAGCGATGCGCTGGGTTGGAGACCACTGGAACTTCCGGTGTCTTCTGGATACCGAAGGTAAGATGGGTCCTAATTGGGCAATTTGCCACTGATACAGGAGGCTACTCATGAACGAAAGACACTTAACAGGTGCTGCTTCTGAAATGCTAGTAGCCTACAAATTTACCAAAGCTGGGTACACTGTCTATTACCCTATGCTGACTTAGAGTAAAGAGGACTTGGTTGTATGGAAGGATTGATGA                                                                                                                                                                                                                                                                                               >  1:57334/1‑301`  
                 `AATCCAAGTAGGCTGCCGTACCGAAGAGATTGCTCAGGTGGTCATTGAGACCGCACAAGAAGCGATGCGCTGGGTTGGAGACCACTGGAACTTCCGGTGTCTTCTGGATACCGAAGGCAAGATGGGTCCTAATTGGGCGATTTGCCACTGATACAGGAGGCTACTCATGAACGAAAGACACTTAACAGGTGCTGCTTCTGAAATGCTAGTAGCCTACAAATTTACCAAAGCTGGGTACACTGTCTATTACCCTATGCTGACTCAGAGTAAAGAGGACTTGGTTGTATGGAAGGATTGATGA                                                                                                                                                                                                                                                                                               >  1:148735/1‑301`  
                    `CCAAGTAGGCTGCCGTACCGAAGAGATTGCTCAGGTGGTCATTGAGACCGCACAAGAAGCGATGCGCTGGGTTGGAGACCACTGGAACTTCCGGTGTCTTCTGGATACCGAAGGTAAGATGGGTCCTAATTGGGCGATTTGCCACTGATACAGGAGGCTACTCATGAACGAAAGACACTTAACAGGTGCTGCTTCTGAAATGCTAGTAGCCTACAAATTTACCAAAGCTGGGTACACTGTCTATTACCCTATGCTGACTCAGAGTAAAGAGGACTTGGTTGTATGGAAGGATTGATGACAT                                                                                                                                                                                                                                                                                            >  1:241025/1‑301`  
                      `gagacaGCTGCCGTACCGAAGAGATTGCTCAGGTGGTCATTGAGACCGCACAAGAAGCGATGCGCTGGGTTGGAGACCACTGGAACTTCCGGTGTCTTCTGGATACCGAAGGTAAGATGGGTCCTAATTGGGCGATTTGCCACTGATACAGGAGGCTACTCATGAACGAAAGACACTTAACAGGTGCTGCTTCTGAAATGCTAGTAGCCTACAAATTTACCAAAGCTGGGTACACTGTCTATTACCCTATGCTGACTCAGAGTAAAGAGGACTTGGTTGTATGGAAGGATTGATGACATTC                                                                                                                                                                                                                                                                                          <  1:101537/295‑1`  
                          `atgtgtataagagacAGAGATTGCTCAGGTGGTCATTGAGACCGCACAAGAAGCGATGCGTTGGGTTGGAGAACACTGGAACTTCCGGTGTCTTCTGGATACCGAAGGTAAGATGGGTCCTAATGGGGCGATTTGCCACTGATACAGGAGGCTACTCATGAACGAAAGACACTTAACAGGTGCTGTTTCTGAAATGCTAGTAGCCTACAAATTTACCAAAGCTGGGTATACTGTCTATTACCCTATGCTGACTCAGAGTAAAGAGGACTTGGTTGTATGGAAGGATTGATGACATTCGTct                                                                                                                                                                                                                                                                                      <  1:95447/286‑3`  
                          `AGGCTGCCGTACCGAAGAGATTGCTCAGGTGGTCATTGAGACCGCACAAGAAGCGATGCGCTGGGTTGGAGACCACTGGAACTTCCGGTGTCTTCTGGATACCGAAGGTAAGATGGGTCCTAATTGGGCGATTTGCCACTGATACAGGAGGCTACTCATGAACGAAAGACACTTAACAGGTGCTGCTTCTGAAATGCTAGTAGCCTACAAATTTACCAAAGCTGGGTACACTGTCTATTACCCTATGCTGACTCAGAGTAAAGAGGACTTGGTTGTATGGAAGGATTGATGACATTCGTAG                                                                                                                                                                                                                                                                                      >  1:35140/1‑301`  
                               `GCCGTACCGAAGAGATTGCTCAGGTGGTCATTGAGACCGCACAAGAAGCGATGCGCTGGGTTGGAGACCACTGGAACTTCCGGTGTCTTCTGGATACCGAAGGTAAGATGGGTCCTAATTGGGCGATTTGCCACTGATACAGGAGGCTACTCATGAACGAAAGACACTTAACAGGTGCTGCTTCTGAAATGCTAGTAGCCTACAAATTTACCAAAGCTGGGTACACTGTCTATTACCCTATGCTGACTCAGAGTAAAGAGGACTTGGTTGTATGGAAGGATTGATGACATTCGTAGTACGT                                                                                                                                                                                                                                                                                 >  1:105470/1‑301`  
                                `CCGTACCGAAGAGATTGCTCAGGTGGTCATTGAGACCGCACAAGAAGCGATGCGCTGGGTTGGAGACCACTGGAACTTCCGGTGTCTTCTGGATACCGAAGGTAAGATGGGTCCTAATTGGGCGATTTGCCACTGATACAGGAGGCTACTCATGAACGAAAGACACTTAACAGGTGCTGCTTCTGAAATGCTAGTAGCCTACAAATTTACCAAAGCTGGGTACACTGTCTATTACCCTATGCTGACTCAGAGTAAAGATGACTTGGTTGTATGGAAGGATTGATGACATTCGTAGTACGTA                                                                                                                                                                                                                                                                                <  1:17863/301‑1`  
                                  `gagacagAGAGATTGCTCAGGTGGTCATTGAGACCGCACAAGAAGCGATGCGCTGGGTTGGAGACCACTGGAACTTCCGGTGTCTTCTGGATACCGAAGGTAAGATGGGTCCTAATTGGGCGATTTGCCACTGATACAGGAGGCTACTCATGAACGAAAGACACTTAACAGGTGCTGCTTCTGAAATGCTAGTAGCCTACAAATTTACAAAAGCTGGGTACACTGTCTATTACCCTATGCTGACTCAGAGTAAAGAGGACTTGGTTGTATGGAAGGATTGATGACATTCGTAGTACGTACA                                                                                                                                                                                                                                                                              <  1:190051/294‑1`  
                                    `ACCGAAGAGATTGCTCAGGTGGTCATTGAGACCGCACAAGAAGCGATGCGCTGGGTTGGAGACCACTGGAACTTCCGGTGTCTTCTGGATACCGAAGGTAAGATGGGTCCTAATTGGGCGATTTGCCACTGATACAGGAGGCTACTCATGAACGAAAGACACTTAACAGGTGCTGCTTCTGAAATGCTAGTAGCCTACAAATTTACCAAAGCTGGGTACACTGTCTATTACCCTATGCTGACTCAGAGTAAAGAGGACTTGGTTGTATGGAAGGATTGATGACATTCGTAGTACGTACATC                                                                                                                                                                                                                                                                            <  1:127443/301‑1`  
                                              `TTGCTCAGGTGGTCATTGAGACCGCACAAGAAGCGATGCGCTGGGTTGGAGACCACTGGAACTTCCGGTGTCTTCTGGATACCGAAGGTAAGATGGGTCCTAATTGGGCGATTTGCCACTGATACAGGAGGCTACTCATGAACGAAAGACACTTAACAGGTGCTGCTTCTGAAATGCTAGTAGCCTACAAATTTACCAAAGCTGGGTACATGGTTATTTACCCTATCCTGACTCAAAGTAAAGAGGATTTGGTTGTATGGAAGGATGGATGACTTCGGTGGTACGTACATCCTTcctctta                                                                                                                                                                                                                                                                  >  1:119179/1‑294`  
                                                `GCTCAGGTGGTCATTGAGACCGCACAAGAAGCGATGCGCTGGGTTGGAGACCACTGGAACTTCCGGTGTCTTCTGGATACCGAAGGTAAGATGGGTCCTAATTGGGCGATTTGCCACTGATACAGGAGGCTACCCATGAACGAAAGACACTTAACAGGTGCTGCTTCTGAAATGCTAGTAGCCTACAAATTTACCAAAGCTGGGTACACTGTCTATTACCCTATGCTGACTCAGAGTAAAGAGGACTTGGTTGTATGGAAGGATTGATGACATTCGTAGGACGTACATCATTTCGTTAGGC                                                                                                                                                                                                                                                                >  1:239860/1‑301`  
                                                   `CAGGTGGTCATTGAGACCGCACAAGAAGCGATGCGCTGGGTTGGAGACCACTGGAATTTCCGGTGTCTTCTGGATACCGAAGGTAATATGGGTCGTAATTGGGCGATTTGCCACTGATACAGGAGGCTACTCATGAACGAAAGACACTTAACAGGTGCTGCTTCTGAAATGCTAGTAGCCTACAAATTTACCAAAGCTGGGTACACTGCCTATTACCATATGCTGACACAGAGTAAAGAGGACTTGGTTGTATGGAAGGATTGATGACATTCGTAGTACGTACATCATTTCGTGAGGCAAT                                                                                                                                                                                                                                                             >  1:162749/1‑301`  
                                                   `CAGGTGGTCATTGAGACCGCACAAGAAGCGATGCGCAGGGTTGGAGACCACTGGAACTTCCGGTGTCTTCTGGATACCGAAGGTAAGATGGGTCCTAATTGGGCGATTTGCCACTGATACAGGAGGCTACTCATGAACGCAAGACACTTAACAGATGCTGCTTCTGAAATGCTCGTAGCCTACAAATTTACCAAAGCTGGGTACACTGTCTATTACCCTATGCTGACTCAGAGTAAAGAGGACTTGGTTGTATGGAAGTATTGATGACATTCTTAGTACGTACATCATTTCGTGAGCCCAT                                                                                                                                                                                                                                                             >  1:91596/1‑301`  
                                                     `gggGGTCAGTGAGACCGCACAAGAAGCGATGCGCTGGGTTGGAGACCACTGGAACTTCCGGTGTCTTCTGGATACCGAAGGTAAGATGGGTCCTAATTGGGCGATTTGCCACTGATACAGGAGGCTACTCATGAACGAAAGACACTTAACAGGTGCTGCTTCTGAAATGCTAGTAGCCTACAAATTTACCAAAGCTGGGTACACTGTCTATTACCCTATGCTGACTCAGAGTAAAGAGGACTTGGTTGTATGGAAGGATTGATGACATTCGTAGTACGTACATCATTTCGTGAGGCCATTA                                                                                                                                                                                                                                                           <  1:185094/298‑1`  
                                                        `GGTCATTGAGACCGCACAAGAAGCGATGCGCTGGGTTGGAGACCACTGGAACTTCCGGTGTCTTCTGGATACCGAAGGTAAGATGGGTCCTAATTGGGCGATTTGCCACTGATACAGGAGGCTACTCATGAACGAAAGACACTTAACAGGTGCTGCTTCTGAAATGCTAGTAGCCTACAAATTTACCAAAGCTGGGTACACTGTCTATTACCCTATGCTGACTCAGAGTAAAGAGGACTTGGTTGTATGGAAGGATTGATGACATTCGTAGTACGTACATCATTTCGTGAGGCCATTAAGG                                                                                                                                                                                                                                                        <  1:159104/301‑1`  
                                                         `agCATTGAGACCGCACAAGAAGCGATGCGCTGGGTTGGAGACCACTGGAACTTCCGGTGTCTTCTGGATACCGAAGGTAAGATGGGTCCTAATTGGGCGATTTGCCACTGATACAGGAGGCTACTCATGAACGAAAGACACTTAACAGGTGCTGCTTCTGAAATGCTAGTAGCCTACAAATTTACCAAAGCTGGGTACACTGTCTATTACCCTATGCTGACTCAGAGTAAAGAGGACTTGGTTGTATGGAAGGATTGATGACATTCGTAGTACGTACATCATTTCGTGAGGCCATTAAGGA                                                                                                                                                                                                                                                       <  1:40377/299‑1`  
                                                           `CATTGAGACCGCACAAGAAGCGATGCGCTGGGTTGGAGACCACTGGAACTTCCGGTGTCTTCTGGATACCGAAGGTAAGATGGGTCCTAATTGGGCGATTTGCCACTGATACAGGAGGCTACTCATGAACGAAAGACACTTAACAGGTGCTGCTTCTGAAATGCTAGTAGCCTACAAATTTACCAAAGCTGGGTACACTGTCTATTACCCTATGCTGACTCAGAGTAAAGAGGACTTGGTTGTATGGAAGGATTGATGACATTCGTAGTACGTACATCATTTCGTGAGGACATTAAGctgt                                                                                                                                                                                                                                                     >  1:214783/1‑297`  
                                                                           `GAAGAGATGCGCTGGGTTGGAGACCACTGGAACTTCCGGTGTCTTCTGGATACCGAAGGTAAGATGGGTCCTAATTGGGCGATTTGCCACTGATACAGGAGGCAACTCATGAACGAAAGACACTTAACAGGTGCTGCTTCTGAAATGCTAGTAGCCTACAAATTTACCAAAGCTGGGTACACTGTCTATTACCCTATGCTGACTCAGAGTAAAGAGGACTTGGTTGTATGGAAGGATTGATGACATTCGTAGTACGTACATCATTTCGTGAGGCCATTAAGGACATGCACGAAGAGTATGC                                                                                                                                                                                                                                     <  1:320757/301‑1`  
                                                                                        `GGGTTGGAGACCACTGGAACTTCCGGTGTCTTCTGGATACCGAAGGTAAGATGGGTCCTAATTGGGCGATTTGCCACTGATACAGGAGGCTACTCATGAACGAAAGACACTTAACAGGTGCTGCTTCTGAAATGCTAGTAGCCTACAAATTTACCAAAGCTGGGTTCACTGTCTATTACCCTATGCTGACTCAGAGTAAAGAGGACTTGGTTGTATGGAAGGATTGATGACATTCGTAGTACGTACATCATTTCGTGAGGCCATTAAGGACATGCACGAAGAGTATGCAGATAAGGACTCT                                                                                                                                                                                                                        >  1:278464/1‑301`  
                                                                                        `GGGTTGGAGACCACTGGAACTTCCGGTGTCTTCTGGATACCGAAGGTAAGATGGGTCCTAATTGGGCGATTTGCCACTGATACAGGAGGCTACTCATGAACGAAAGACACTTAACAGGTGCTGCTTCTGAAATGCTAGTAGCCTACAAATTTACCAAAGCTGGGTACACTGTCTATTACCCTATGCTGACTCAGAGTAAAGAGGACTTGGTTGTATGGAAGGATTGATGACATTCGTAGTACGTACATCATTTCGTGAGGCCATTAAGGACATGCACGAAGAGTATGCAGATAAGGACTCT                                                                                                                                                                                                                        >  1:113162/1‑301`  
                                                                                             `GGAGACCACTGGAACTTCCGGTGTCTTCTGGATACCGAAGGTAAGATGGGTCCTAATTGGGCGATTTGCCACTGATACAGGAGGCTACTCATGAACGAAAGACACTTAACAGGTGCTGCTTCTGAAATGCTAGTAGCCTACAAATTTACCAAAGCTGGGTACACTGTCTATTACCCTATGCTGACTCAGAGTAAAGAGGACTTGGTTGTATGGAAGGATTGATGACATTCGTAGTACGTACATCATTTCGTGAGGCCATTAAGGACATGCACGAAGAGTATGCAGATAAGGACTCTTTCAA                                                                                                                                                                                                                   >  1:145969/1‑301`  
                                                                                               `atgtgtataagagacagGGTGTCTTCTGGATACCGAAGGTAAGATTGGGCCTAATTGGGCGATTTGCCACTGATACAGGAGGCTACTCATGAACGAAAGACACTTAACAGGTGCTGCTTCTGAAATGCTAGTAGCCTACAAATTTACCAAAGCTGGGTACACTGTCTATTACCCTATGCTGACTCAGAGTAAAGAGGACTTGGTTGTATGGAAGGATTGATGACATTCGTAGTACGTACATCATTTCGTGAGGCCATTAAGGACATGCACGAAGAGTATGCAGATAAGGACTCTTTCAAAC                                                                                                                                                                                                                 <  1:325505/284‑1`  
                                                                                               `AGACCACTGGAACTTCCGGTGTCTTCTGGATACCGAAGGTAAGATTGGTCCTAATTGGGCGATTTGCCACTGATACAGGAGGCTACTCATGAACGAAAGACACTTAACAGGTGCTGCTTCTGAAATGCTAGTAGCCTACAAATTTACCAAAGCTGGGTACACTGTCTATTACCCTATGCTGACTCAGAGTAAAGAGGACTTGGTTGTATGGAAGGATTGATGACATTCGTAGTACGTACATCATTTCGTGAGGCCATTAAGGACATGCACGAAGAGTATGCAGATAAGGACTCTTTCAAAC                                                                                                                                                                                                                 <  1:68580/301‑1`  
                                                                                               `AGACCACTGGAACTTCCGGTGTCTTCTGGATACCGAAGGTAAGATGGGTCCTAATTGGGCGATTTGCCACTGATACAGGAGGCTACTCATGAACGAAAGACACTTAACAGGTGCTGATTCTGAAATGCTAGTAGCCTACAAATTTACCAAAGCTGGGTACACTGTCTATTACCCTATGCTGACTCAGAGTAAAGAGGACTTGGTTGTATGGAAGGATTGATGACATTCGTAGTACGTACATCATTTCGTGAGGCCATTAAGGACATGCACGAAGAGTATGCAGATAAGGACTCTTTCAAAC                                                                                                                                                                                                                 <  1:89801/301‑1`  
                                                                                                                `GGTTTCTTCTGGATACCGAAGGTAAGATGGGTCCTAATTGGGCGATTTGCCACTGATACAGGAGGCTACTCATGAACGAAAGACACTTAACAGGTGCTGCTTCTGAAATGCTAGTAGCCTACCAATTTACCAAAGCTGGGTACACTGTCTATTACCCTATGCTGACTCAGAGTAAAGAGGACTTGGTTGTATGGAAGGATTGATGACATTCGTAGTACGTACATCATTTCGTGAGGCCATTAAGGACATGCACGAAGAGTATGCAGATAAGGACTCTTTCAAAAAATCTCCTGCAACATTA                                                                                                                                                                                                >  1:115591/1‑301`  
                                                                                                                 `GTGTCTTCTGGATACCGAAGGTAAGATGGGTCCTAATTGGGCGATTTGCCACTGATACAGGAGGCTACTCATGAACGAAAGACACTTAACAGGTGCTGCTTCTGAAATGCTAGTAGCCTACAAATTTACCAAAGCTGGGTACACTGTCTATTACCCTATGCTGACTCAGAGTAAAGAGGACTTGGTTGTATGGAAGGATTGATGACATTCGTAGTACGTACATCATTTCGTGAGGCCATTAAGGACATGCACGAAGAGTATGCAGATAAGGACTCTTTCAAACAATCTCCTGCAACAGTAC                                                                                                                                                                                               >  1:110641/1‑301`  
                                                                                                                   `GTCTTCTGGATACCGAAGGTAAGATGGGTCCTAATTGGGCTATTTGCCACTGATACAGGAGGCTACTCATGAACGAAAGACACTTAACAGGTGCTGCTTCTGAAATGCTAGTAGCCTACAAATTTACCAAAGCTGGGTACACTGTCTATTACCCTCTGCTGACTCAGAGTAAAGAGGACTTGGTTGTATGGAAGGATTGATAACATTCGTAGTACGTACATCATTTCGTGAGTCCTTTAAGGAACTGCAAGAAGAGTATGAAGATAAGGCCTATTTCAAACAATCTACTGCAACAGTcagg                                                                                                                                                                                             >  1:27285/1‑297`  
                                                                                                                   `GTCTTCTGGATACCGAAGGTAAGATGGGTCCTAATTGGGCGATTTGCCACTGATACAGGAGGCTACTCATGAACGAAAGACACTTAACAGGTGCTGCTTCTGAAATGCTAGTAGCCTACAAATTTACCAAAGCTGGGTACACTGTCTATTACCCTATGCTGACTCAGAGTAAAGAGGACTTGGTTGTATGGAAGGATTGATGACATTCGTAGTACGTACATCATTTCGTGAGGCCATTAAGGACATGCACGAAGAGTATGCAGATAAGGACctgtctcttatacacatctccgagcccacg                                                                                                                                                                                             >  1:132381/1‑271`  
                                                                                                                     `CTTCTGGATACCGAAGGTAAGATGGGTCCTAATTGGGCGATTTGACACTGATACAGGAGGCTACTCATGAACGAAAGACACTTAACAGGTGCTGCTTCTGAAATGCTAGTAGACTACAAATTTACCAAAGCTGGGTACACTGTCTATTACCCTATGCTGACTCAGAGTAAAGAGGACTTGGTTGTATGGAAGGATTGATGACATTCGTAGTACGTACATCATTTCGTGAGGCCATTAAGGACATGCACGAAGAGTATGCAGATAAGGACTCTTTCAAACAATCTCCTGCAACAGTACGGGA                                                                                                                                                                                           >  1:85153/1‑301`  
                                                                                                                       `TCTGGATACCGAAGGTAAGATGGGTCCTAATTGGGCGATTTGCCACTGATACAGGAGGCTACTCATGAACGAAAGACACTTAACAGGTGCTGCTTCTGAAATGCTAGTAGCCTACAAATTTACCAAAGCTGGGTACACTGTCTATTACCCTATGCTGACTCAGAGTAAAGAGGACTTGGTTGTATGGAAGGATTGATGACATTCGTAGTACGTACATCATTTCGTGAGGCCATTAAGGACATGCACGAAGAGTATGCAGATAAGGACTCTTTCAAACAATCTCCTGCAACAGctgtctctt                                                                                                                                                                                         >  1:45497/1‑292`  
                                                                                                                       `TCTGGATACCGAAGGTAAGATGGGTCCTAATTGGGCGATTTGCCACTGATACAGGAGGCTACTCATGAACGAAAGACACTTAACAGGTGCTGCTTCTGAAATGCTAGTAGCCTACAAATTTACCAAAGCTGGGTACACTGTCTATTACCCTATGCTGACTCAGAGTAAAGAGGACTTGGTTGTATGGAAGGATTGATGACATTCGTAGTACGTACATCATTTCGTGAGGCCATTAAGGACATGCACGAAGAGTATGCAGATAAGGACTCTTTCAAACAATCTCCTGCAACAGTACGGGAGG                                                                                                                                                                                         >  1:54870/1‑301`  
                                                                                                                             `TACCGAAGGTAAGATGGGTCCTAATTGGGCGATTTGCCACTGATACAGGAGGCTACTCATGAACGAAAGACACTTAACAGGTGCTGCTTCTGAAATGCTAGTAGCCTACAAATTTACCAAAGCTGGGTACACTGTCTATTACCCTATGCTGACTCAGAGTAAAGAGGACTTGGTTGTATGGAAGGATTGATGACATTCGTAGTACGTACATCATTTCGTGAGGCCATTAAGGACATGCACGAAGAGTATGCAGATAAGGACTCTTTCAAACTGTCTCttatacacatctccgagcccacgg                                                                                                                                                                                   >  1:246726/1‑277`  
                                                                                                                               `CCGAAGGTAAGATAGGTCCTAATTGGGCGATTTGCCACTGATACAGGAGGCTACTCATGAACGAAAGACACTTAACAGGTGCTGCTTCTGTAATGCTAGTCACCTACAAATTTACCAAAGCTGGGTACACTGTCTATTACCCTATGCTGACTCAGAGTAAAGAGGACATGGTTGTATGGAAGGATTGATGACATTCGTAGTACGTACATCATTTCGTGAGGCCATAAAGGACATGCACGAAGAGTATGCAGATAAGGACTCTTTCAAACAATCTCCTGCAACAGTACGGGAGGTGTTCTGA                                                                                                                                                                                 <  1:163712/301‑1`  
                                                                                                                                    `GGTAAGATGGGTCCTAATTGGGCGATTTGCCACTGATACAGGAGGCTACTCATGAACGAAAGACACTTAACAGGTGCTGCTTCTGAAATGCTAGTAGCCTACAAATTTACCAAAGCTGGGTACACTGTCTATTACCCTATGCTGACTCAGAGTAAAGAGGACTTGGTTGTATGGAAGGATTGATGACATTCGTAGTACGTACATCATTTCGTGAGGCCATTAAGGACATGCACGAAGAGTATGCAGATAAGGACTCTTTCAAACAATCTCCTGCAACAGTACGGGAGGTGTTCTGATGTCT                                                                                                                                                                            >  1:287368/1‑301`  
                                                                                                                                             `GGTCCTAATTGGGCGATTTGCCACTGATACAGGAGGCTACTCATGAACGAAAGACACTTAACAGGTGCTGCTTCTGAAATGCTAGTAGCCTACAAATTTACCAAAGCTGGGTACACTGTCTATTACCCTATGCTGACTCAGAGTAAAGAGGACTTGGTTGTATGGAAGGATTGATGACATTCGTAGTACGTAAATCATTTCGTGAGGCCATTAAGGACATGCACGAAGAGTATGCAGATAAGGACTCTTTCAAACAATCTCCTGCAACAGTACGGGAGGTGTTCTGATGTCTGACTACATG                                                                                                                                                                   >  1:47879/1‑301`  
                                                                                                                                                                     `aaagagacagGGCTACTCATGAAAGAAAGACACTTAACAGGTGCTGCTTCTGAAATGCTAGTAGCCTACAAATTTACCAAAGCTGGGTACACTGTCTATTACCCTATGCTGACTCAGAGTAAAGAGGACTTGGTTGAATGGAAGGATTGATGACATTCGTAGTACGTACATCATTTCGTGAGGCCATTAAGGACATGCACGAAGAGTATGCAGATAAGGACTCTTTCAAACAATCTCCTGCAACAGTACGGGAGGTGTTCTGATGTCTGACTACCTGAAAGTGCTGCAAGCAATCAAAAGT                                                                                                                                           <  1:187078/291‑1`  
                                                                                                                                                                         `tgggctcggagatgtgtataagagacagCTTAACAGGTGCTGCTTCTGAAATGCTAGTAGACTACAAATTTACCAAAGCTGGGTACACTGTCTATTACCCTATGCTGACTCAGAGTAAAGAGGACTTGGTTGTATGGAAGGATTGATGACATTCGTAGTACGTACATCATTTCGTGAGGCCATTAAGGACATGCACGAAGAGTATGCAGATAAGGACTCTTTCAAACAATCTCCTGCAACAGTACGGGAGGTGTTCTGATGTCTGACTACCTGAAAGTGCTGCAAGCAATCAAAAGTTGCC                                                                                                                                       <  1:43403/273‑1`  
                                                                                                                                                                               `GGCTACTCATGAACGAAAGACACTTAACAGGTGCTGCTTCTGAAATGCTAGTAGCCTACAAATTTACCAAAGCTGGGTACACTGTCTATTACCCTATGCTGACTCAGAGTAAAGAGGACTTGGTTGTATGGAAGGATTGATGACATTCGTAGTACGTACATCATTTCGTGAGGCCATTAAGGACATGCACGAAGAGTATGCAGATAAGGACTCTTTCAAACAATCTCCTGCAACAGTACGGGAGGTGTTCTGATGTCTGACTACCTGAAAGTGCTGCAAGCAATCAAAAGTTGCCCTAAGA                                                                                                                                 >  1:135900/1‑301`  
                                                                                                                                                                               `GGCTACTCATGAACGAAAGACACTTAACAGGTGCTGCTTCTGAAATGCTAGTAGCCTACAAATTTACCAAAGCTGGGTACACTGTCTATTACCCTATGCTGACTCAGAGTAAAGAGGACTTGGTTGTATGGAAGGATTGATGACATTCGTAGTACGTACATCATTTCGTGAGGCCATTAAGGACATGAACGAAGAGTATGCAGATAAGGACTCTTTCAAACAATCTCCTGCAACAGTACGGGAGGTGTTCTGATGTCTGACTACCTGAAAGTGCTGCAAGCAATCAAAAGTctgtctctta                                                                                                                                 >  1:331703/1‑291`  
                                                                                                                                                                                `GCTACTCATGAACGAAAGACACTTAACAGGTGCTGCTTCTGAAATGCTAGTAGCCTACAAATTTACCAAAGCTGGGTACACTGTCTATTACCCTATGCTGACTCAGAGTAAAGAGGACTTGGTTGTATGGAAGGATTGATGACATTCGTAGTACGTACATCATTTCGTGAGGCCATTAAGGACATGCACGAAGAGTATGCAGATAAGGACTCTTTCAAACAATCTCCTGCAACAGTACGGGAGGTGTTCTGATGTCTGACTACCTGAAAGTGCTGCAAGCAATCAAAAGTTGCCCTAAGAC                                                                                                                                <  1:265306/301‑1`  
                                                                                                                                                                                `GCTACTCATGAACGAAAGACACTTAACAGGTGCTGCTTCTGAAATGCTAGTAGCCTACAAAATTACCAAAGCTGGGTACACTGTCTATTACCCTATGCTGAATCAGAGTAAAGAGGACTTGGTTGTATGGAAGGATTGATGACATTCGTAGTACGTACATCATTTCGTGAGGCCATTAAGGACATGCACGAAGAGTATGCAGATCAGGACTCTTTCAAACAATCTCCTGCAACAGTACGGGAGGTGTTCTGATGTCTGACTACCTGGAAGTGCTGCAAGCAATCAAAAGTTGCCCTAAGAC                                                                                                                                <  1:53237/301‑1`  
                                                                                                                                                                                `GCTACACATGAACGAAAGACACTTAACAGGTGCTGCTTCTGAAATGCTAGTAGCCTACAAATTTACGAAAGCTGGATACACTGTCTATTACCCTATGCTGACTCAGAGTAAAGAGGACTTGGTTGTACGGAAGGATTGATGACATTCGTAGTACGTACATCATTTCGTGAGGCCATTAAGGACATGTACGAAGAGTATGCAGATAAGGACTCTTTCAAACAATCTCCTGCAACAGTACGGGAGGTGTTCTGATGTCTGACTACCTGAAAGTGCTGCAAGCAATCAAAAGTGGCCCTAAGAC                                                                                                                                <  1:202743/301‑1`  
                                                                                                                                                                                    `CTCATGAACGAAAGACACTTAACAGGTGCTGCTTCTGAAATGCTAGTAGCCTACAAATTTACCAAAGCTGGGTACACTGTCTATTACCCTATGCTGACTCAGAGTAAAGAGGACTTGGTTGTATGGAAGGATTTATGACATTCGTAGTACGTACATCATTTCGTGAGGCCATTAAGGACATGCACGAAGAGTATGCAGATAAGGACTCTTTCAAACAATCTCCTGCAACAGTACGGGAGGTGTTCTGATGTCTGACTACCTGAAAGTGCTGCAAGCAATCAAAAGTTGCCCTAAGACTTTC                                                                                                                            >  1:295696/1‑301`  
                                                                                                                                                                                     `TCATGAACGAAAGACACTTAACAGGTGCTGCTTCTGAAATGATAGTAGCCTACAAATTGACCAAAGCTGGGTACACTGTCTATTACCCTATGCTGACTCAGAGTAAAGAGGACTTGGTTGTATGGAAGGATTGATGACATTCGTAGTACGGACATCATTTCGTGAGGCCATTAAGGACATGCACGAAGAGTATGCAGATAAGGACTCTTTCAAACAATCTCCTGCAACAGTACGGGAGGTGTTCTGATGTCTGACTACCTGAAAGTGCTGCAAGCAATCAAAAGTTGCCCTAAGACTTTCC                                                                                                                           <  1:304269/301‑1`  
                                                                                                                                                                                               `AAGACACTTAACAGGTGCTGCTTCTGAAATGCTAGTAGCCTACAAATTTACCAAAGCTGGGTACACTGTCTATTACCCTATGCTGACTCAGAGTAAAGAGGACTTGGTTGTATGGAAGGATTGATGACATTCGTAGTACGTACATCATTTCGTGAGGCCATTAAGGACATGCACGAAGAGTATGCAGATAAGGACTCTTTCAAACAATCTCCTGCAACAGTACGGGAGGTGTTCTGATGTCTGACTACCTGAAAGTGCTGCAAGCAATCAAAAGTTGCCCTAAGACTTTCCAGTCCAACTA                                                                                                                 <  1:154636/301‑1`  
                                                                                                                                                                                                   `CACTTAACAGGTGCTGCTTCTGAAATGCTAGTAGCCTACAAATTTACCAAAGCTGGGTACACTGTCTATTACCCTATGCTGACTCAGAGTAAAGAGGACTTGGTTGTATGGAAGGATTGATGACATTCGTAGTACGTACATCATTTCGTGAGGCCATTAAGGACATGCACGAAGAGTATGCAGATAAGGACTCTTTCAAACAATCTCCTGCAACAGTACGGGAGGTGTTCTGATGTCTGACTACCTGAAAGTGCTGCAAGCAATCAAAAGTTGCCCTAAGACTTTCCAGTCCAACTATGTA                                                                                                             >  1:316333/1‑301`  
                                                                                                                                                                                                    `ACTTAACAGGTGCTGCTTCTGAAATGCTAGTAGCCTACAAATTTACCAAAGCTGGGTACACTGTCTATTACCCTATGCTGACTCAGAGTAAAGAGGACTTGGTTGTATGGAAGGATTGATGACATTCGTAGTACGTACATCATTTCGTGAGGCCATTAAGGACATGCACGAAGAGTATGCAGATAAGGACTCTTTCAAACAATCTCCTGCAACAGTACGGGAGGTGTTCTGATGTCTGACTACCTGAAAGTGCTGCAAGCAATCAAAAGCTGtctcttatacacatctccgagcccacgag                                                                                                            >  1:75701/1‑272`  
                                                                                                                                                                                                     `CTTAACAGGTGCTGCTTCTGAAATGCTAGTAGCCTACAAATTTACCAAAGCTGGGTACACTGTCTATTACCCTATGCTGACTCAGAGTAAAGAGGACTTGGTTGTATGGAAGGATTGATGACATTCGTAGTACGTACATCATTTCGTGAGGCCATTAAGGACATGCACGAAGAGTATGCAGATAAGGACTCTTTCAAACAATCTCCTGCAACAGTACGGGAGGTGTTCTGATGTCTGACTACCTGAAAGTGCTGCAAGCAATCAAAAGTTGCCCTAAGACTTTCCAGTCCAACTATGTACG                                                                                                           >  1:290173/1‑301`  
                                                                                                                                                                                                       `TAACAGGTGCTGCTTCTGAAATGCTAGTAGCCTACAAATTTACCAAAGCTGGGTACACTGTCTATTACCCTATGCTGACTCAGAGTAAAGAGGACTTGGTTGTATGGAAGGATTGATGACATTCGTAGTACGTACATCATTTCGTGAGGCCATTAAGGACATGCACGAAGAGTATGCAGATAAGGACTCTTTCAAACAATCTCCTGCAACAGTACGGGAGGTGTTCTGATGTCTGACTACCTGAAAGTGCTGCAAGCAATCAAAAGTTGCCCTAAGACTTTCCAGTCCAACTATGTACGGA                                                                                                         <  1:334580/301‑1`  
                                                                                                                                                                                                         `ACAGGTGCTGCTTCTGAAATGGTAGTAGCCTACAAATTTACCAAAGCTGGGTACACTGTCTATTACCCTATGCTGACTCAGAGTAAAGAGGACTTGGTTGTATGGAAGGAGTGAGGACATTCGTAGTACGTACATAATTTCGTGAGGACATTAAGGACATGCACGAAGAGTATGCAGATAAGGACTCTTTCAAACAATCTCCTGCAACAGTACGGGAGGTGTTCTGATGTCTGACTACCTGAAAGTGCTGCAAGCAATCAAAAGTTGACCTAAGACTTTCCAGTCCAACTATGTACGGAAC                                                                                                       <  1:108142/301‑1`  
                                                                                                                                                                                                         `ACAGGTGCTGCTTCTGAAATGCTAGTAGCCTACAAATTTACCAAAGCTGGGTACACTGTCTATTACCCTATGCTGACTCAGAGTAAAGAGGACTTGGTTGTATGGAAGGATTGATGACATTCGTAGTACGTACATCATTTCGTGAGGCCATTAAGGACATGCACGAAGAGTATGCAGATAAGGACTCTTTCAAACAATCTCCTGCAACAGTACGGGAGGTGTTCTGATGTCTGACTACCTGAAAGTGCTGCAAGCAATCAAAAGTTGCCCTAAGACTTTCCAGTCCAACTATGTACGGAAC                                                                                                       <  1:79212/301‑1`  
                                                                                                                                                                                                             `GTGCTGCTTCTGAAATGCTAGTAGCCTACAAATTTACCAAAGCTGGGTACACTGTCTATTACCCTATGCTGACTCAGAGTAAAGAGGACTTGGTTGTATGGAAGGATTGATGACATTCGTAGTACGTACATCATTTCGTGAGGCCATTAAGGACATGCACGAAGAGTATGCAGATAAGGACTCTTTCAAACAATCTCCTGCAACAGTACGGGAGGTGTTCTGATGTCTGACTACCTGAAAGTGCTGCAAGCAATCAAAAGTTGCCCTAAGACTTTCCAGTCCAACTATGTACGGAACAATG                                                                                                   >  1:217326/1‑301`  
                                                                                                                                                                                                                                         `ctacTTTACCAAAGCTGGGTACACTGTCTATTACCCTATGCTGACTCAGAGTAAAGTGGACTTGGTTGTATGGAAGGATTTATGACATTCGTAGTACGTACATCATTTCGTGAGGCCATTAAGGACATGCACGAAGAGTATGCAGATAAGGACTCTTTCAAACAATCTCCTGCAACAGTACGGGAGGTGTTCTGATGTCTGACTACCTGTAAGTGCTGCAAGCAATCAAAAGTTGCCCTAAGACTTTCCAGTCCAACTATGTACGGAACAATGCGAGCCTCGTAGCGGAGGCCGCTTCCCG                                                                       <  1:3731/297‑1`  
                                                                                                                                                                                                                                           `AATTTACCAAAGCTGGGTACACTGTCTATTACCCTATGCTGACTCAGAGTAAAGAGGACTTGGTTGTATGGAAGGATTGATGACATTCGTAGTACGTACATCATTTCGTGAGGCCATTAAGGACATGCACGAAGAGTATGCAGATAAGGACTCTTTCAAACAATCTCCTGCAACAGTACGGGAGGTGTTCTGATGTCTGACTACCTGAAAGTGCTGCAAGCAATCAAAAGTTGCCCTAAGACTTTCCAGTCCAACTATGTACGGAACAATGCGAGCCTCGTAGCGGAGGCCGCTTCCCGTG                                                                     <  1:60460/301‑1`  
                                                                                                                                                                                                                                           `AATTTACCAAAGCTGGGTACACTGTCTATTACCCTATGCTGACTCAGAGTAAAGAGGACTTGGTTGTATGGAAGGATTGATGACATTCGTAGTACGTACATCATTTCGTGAGGCCATTAAGGACATGCACGAAGAGTATGCAGATAAGGACTCTTTCAAACAATCTCCTGCAACAGTACGGGAGGTGTTCTGATGTCTGACTACCTGAAAGTGCTGCAAGCAATCAAAAGTTGCCCTAAGACTTTCCAGTCCAACTATGTACGGAACAATGCGAGCCTCGTAGCGGAGGCCGCTTCCCGTG                                                                     >  1:260469/1‑301`  
                                                                                                                                                                                                                                                    `AAGCTGGGTACACTGTCGATTACCCTATGCTGACTCAGAGTAAAGAGGACTTGGTTGTATGGAAGGATTGATGACATTCGTAGTACGTACATCATTTCGTGAGGCCATTAAGGACATGCACGAAGAGTATGCAGATAAGGACTCTTTCAAACAATCTCCTGCAACAGTACGGGAGGTGTTCTGATGTCTGACTACCTGAAAGTGCTGCAAGCAATCAAAAGTTGCCCTAAGACTTTCCAGTCCAACTATGTACGGAACAATGCGAGCCTCGTAGCGGAGGCCGCTTCCCGTGGTCACATCT                                                            <  1:65240/301‑1`  
                                                                                                                                                                                                                                                      `GCTGGGTACACTGTCTATTACCCTATGCTGACTCAGAGTAAAGAGGACTTGGTTGTATGGAAGGATTGATGACATTCGTAGTACGTACATCATTTCGTGAGGCCATTAAGGACATGCACGAAGAGTATGCAGATAAGGACTCTTTCAAACAATCTCCTGCAACAGTACGGGAGGTGTTCTGATGTCTGACTACCTGAAAGTGCTGCAAGCAATCAAAAGTTGCCCTAAGACTTTCCAGTCCAACTATGTACGGAACAATGCGAGCCTAGTAGCGGAGGCCGCTTCCCGTTGTCACATCTCG                                                          >  1:329937/1‑301`  
                                                                                                                                                                                                                                                          `gtatataagagacagACCCTATGCTGACTCAGAGGAAAGAGGACTTGGTTGTATGGAAGGATTGATGACATTCGTAGTACGTACATCATTTCGTGAGGCCATTAAGGACATGCACGAAGAGTATGCAGATAAGGACTCTTTCAAACAATCTCCTGCAACAGTACGGGAGGTGTTCTGATGTCTGACTACCTGAAAGTGCTGCAAGCAATCAAAAGTTGCCCTAAGACTTTCCAGTCCAACTATGTACGGAACAATGCGAGCCTCGTAGCGGAGGCCGCTTCCCGTGGTCACATCTCGTGCC                                                      <  1:151842/286‑1`  
                                                                                                                                                                                                                                                                  `GTCTATTACCCTATGCTGACTCAGAGTAAAGAGGACTTGGTTGTATGGAAGGATTGATGACATTCGTAGTACGTACATCATTTCGTGAGGCCATTAAGGACATGCACGAAGAGTATGCAGATAAGGACTCTTTCAAACAATCTCCTGCAACAGTACGGGAGGTGTTCTGATGTCTGACTACCTGAAAGTGCTGCAAGCAATCAAAAGTTGCCCTAAGACTTTCCAGTCCAACTATGTACGGAACAATTCGAGCCTCGTAGCGGAGGCCGCTTCCCGTGGTCACATCTCGTGCCTGACTACT                                              >  1:281085/1‑301`  
                                                                                                                                                                                                                                                                  `GTCTATTACCCTATGCTGACTCAGAGTAAAGAGGACTTGGTTGTATGGAAGGATTGATGACATTCGTAGTACGTACATCATTTCGTGAGGCCATTAAGGACATGCACGAAGAGTATGCAGATAAGGACTCTTTCAAACAATCTCCTGCAACAGTACGGGAGGTGTTCTGATGTCTGACTACCTGAAAGTGCTGCAAGCAATCAAAAGTTGCCCTAAGACTTTCCAGTCCAACGATGTACGGAACAATACGAGCTTCGTAGCGGAGGCCGCTTCCCGTGGTCACATCTCGTGCCTAACTACT                                              >  1:192701/1‑301`  
                                                                                                                                                                                                                                                                    `CTATTACCCTATGCTGACTCAGAGTAAAGAGGACTTGGTTGTATGGAAGGATTGATGACATTCGTAGTACGTACATCATTTTGTGAGGCCATTAAGGACATGCCCGAAGAGTATGCTGACAAGGACTCGTTCAAACAATCTCCTGCAATAGTACGGGAGGGGTTCTGATATATGACTACCTGAAAGTGCGTCAAGCAATCAAAAGTTGCCCTAAGACTTTGCAGGCTAACTATGTACGGAATAAAGCGAGCCTCGTCGCGGAGGCCGCTGCCTGTGGTACCTGCTCGTGCCTGTCTGCTAG                                            >  1:206775/1‑301`  
                                                                                                                                                                                                                                                                    `CTATTACCCTATGCTGACTCAGAGTAAAGAGGACTTGGTTGTATGGAAGGATTGATGACATTCGTAGTACGTACATCATTTCGTGAGGCCATTAAGGACATGCACGAAGAGTATGCAGATAAGGACTCTTTCAAACAATCTCCTGCAACAGTACGGGAGGTGTTCTGATGTCTGACTACCTGAAAGTGCTGCAAGCAATCAAAAGTTGCCCTAAGACTTTCCAGTCCAACTATGTACGGAACAATGCGAGCCTCGTAGCGGAGGCCGCTTCCCGTGGTCACATCTCGTGCCTGACTACTAG                                            <  1:13865/301‑1`  
                                                                                                                                                                                                                                                                    `CTATTACCCTATGCTGACTCAGAGTAAAGAGGACTTGGTTGTATGGAAGGATTGATGACATTCGTAGTACGTACATCATTTCGTGAGGCCATTAAGGACATGCACGAAGAGTATGCAGATAAGGACTCTTTCAAACAATCTCCTGCAACAGTACGGGAGGTGTTCTGATGTCTGACTACCTGAAAGTGCTGCAAGCAATCAAAAGTTGCCCTAAGACTTTCCAGTCCAACTATGTACGGAACAATGCGAGCCTCGTAGCGGAGGCCGCTTCCCGTGGTCACATCTCGTGCCTGACTACTAG                                            >  1:134803/1‑301`  
                                                                                                                                                                                                                                                                    `CTATTACCCTATGCTGACTCAGAGTAAAGAGGACTTGGTTGTATGGAAGGATTGATGACATTCGTAGTACGTACATCATTTCGTGAGGCCATTAAGGACATAAAGGAAGAGTATGCAGATAAGGACTCTTTCAAACAATCTCCTGACACAGTACGGGAGGTGTTCTGATGTCTGACTACCTGAAAGTGCTGCAAGCAATCAAAAGTTGCCCTAAGACTTTCCAGTCCAAATATGTACGGCACAATGCGAGCCTCGTAGCGGAGGACGCTTCCCTTTGTCACATCTCGTCCCTGCCGACTAG                                            >  1:50546/1‑301`  
                                                                                                                                                                                                                                                                      `ATTACCCTATGCTGACTCAGAGTAAAGAGGACTTGGTTGTATGGAAGGATTGATGACATTCGTAGTACGTACATCATTTCGTGAGGCCATTAAGGACATGCACGAAGAGTATGCAGATAAGGACTCTTTCAAACAATCTCCTGCAACAGTACGGGAGGTGTTCTGATGTCTGACTACCTGAAAGTGCTGCAAGCAATCAAAAGTTGCCCTAAGACTTTCCAGTCCAACTATGTACGGAACAATGCGAGCCTCGTAGCGGAGGCCGCTTCCCGTGGTCACATCTCGTGCCTGACTACTAGTG                                          <  1:243660/301‑1`  
                                                                                                                                                                                                                                                                      `ATTACCCTATGCTGACTCAGAGTAAAGAGGACTTGGTTGTATGGAAGGATTGATGACATTCGTAGTACGTACATCATTTCGTGAGGCCATTAAGGACATGCACGAAGAGTATGCAGATAAGGACTCTTTCAAACAATCTCCTGCAACAGTACGGGAGGTGTTCTGATGTCTGACTACCTGAAAGTGCTGCAAGCAATCAAAAGTTGCCCTAAGACTTTCCAGTCCAACTATGTACGGAACAATGCGAGCCTCGTAGCGGAGGCCGCTTCCCGTGGTCACATCTCGTGCCTGACTACTAGTG                                          <  1:19884/301‑1`  
                                                                                                                                                                                                                                                                      `ATTACCCTATGCTGACTCAGAGTAAAGAGGACTTGGTTGTATGGAAGGATTGATGACATTCGTAGTACGTACATCATTTCGTGAGGCCATTAAGGACATGCACGAAGAGTATGCAGATAAGGACTCTTTCAAACAATCTCCTGCAACAGTACGGGAGGTGTTCTGATGTCTGACTACCTGAAAGTGCTGCAAGCAATCAAAAGTTGCCCTAAGACTTTCCAGTCCAACTATGTACGGAACAATGCGAGCCTCGTAGCGGAGGCCGCTTCCCGTGGTCACATCTCGTGCCTGACTACTAGTG                                          <  1:30995/301‑1`  
                                                                                                                                                                                                                                                                        `agacagATGCTGACTCAGAGTAAAGAGGACTTGGTTGTATGGAAGGATTGATGACATTCGTAGTACGTACATCATTTCGTGAGGCCATTAAGGACATGCACGAAGAGTATGCAGATAAGGACTCTTTCAAACAATCTCCTGCGACAGTACGGGAGGTGTTCTGATGTCTGACTACCTGAAAGTGCTGCAAGCAATCAAAAGTTGCCCTAAGACTTTCCAGTCCAACTATGTACGGAACAATGCGAGCCTCGTAGCGGAGGCCGCTTCCCGTGGTCACATCTCGTGCCTGACTACTAGTGGA                                        <  1:81504/295‑1`  
                                                                                                                                                                                                                                                                         `ACCCTATTCTGACTCAGAGTAAAGAGGACTTGGTTGTATGGAAGGATTGATGACATTCGTAGTACGTACATCATTTCGTGAGGCCATTAAGGACATTCACGAAGAGTATGCAGATAAGGACTCTTTCAAACAATCTCCTGCAACAGTACGGGAGGTGTTCTGATGTCTGACTACCTGAAAGTGCTGCAAGCAATCAAAAGTTGCCCTAAGACTTTCCAGTCCAACTATGTACGGAACAATGCGAGCCTCGTAGCGGAGGCCGCTTCCCGTGGTCACATCTCTTGCCTGACTACTAGTGGAC                                       >  1:156595/1‑301`  
                                                                                                                                                                                                                                                                         `ACCCTATGCTGACTCAGAGTAAAGAGGACTTGGTTGTATGGAAGGATTGATGACATTCGTAGTACGTACATCATTTCGTGAGGCCATTAAGGACATGCACGAAGAGTATGCAGATAAGGACTCTTTCAAACAATCTCCTGCAACAGTACGGGAGGTGTTCTGATGTCTGACTACCTGAAAGTGCTGCAAGCAATCAAAAGTTGCCCTAAGACTTTCCAGTCCAACTATGTACGGAACAATGCGAGCCTCGTAGCGGAGGCCGCTTCCCGTGGctgtctcttatacacatctccgagcccac                                       >  1:249989/1‑272`  
                                                                                                                                                                                                                                                                         `ACCCTATGCTGACTCAGAGTAAAGAGGACTTGGTTGTATGGAAGGATTGATGACATTCGTAGTACGTACATCATTTCGTGAGGCCATTAAGGACATGCACGAAGAGTATGCAGATAAGGACTCTTTCAAACAATCTCCTGCAACAGTACGGGAGGTGTTCTGATGTCTGACTACCTGAAAGTGCTGCAAGCAATCAAAAGTTGCCCTAAGACTTTCCAGTCCAACTATGTACGGAACAATGCGAGCCTCGTAGCGGAGGCCGCTTCCCGTGGTCACATCTCGTGCCTGACTACTAGctgtc                                       >  1:275073/1‑296`  
                                                                                                                                                                                                                                                                         `ACCCTATGCTGACTCAGAGTAAAGAGGACTTGGTTGTATGGAAGGATTGATGACATTCGTAGTACGTACATCATTTCGTGAGGCCATTAAGGACATGCACGAAGAGTATGCAGATAAGGACTCTTTCAAACAATCTCCTGCAACAGTACGGGAGGTGTTCTGATGTCTGACTACCTGAAAGTGCTGCAAGCAATCAAAAGTTGCCCTAAGACTTTCCAGTCCAACTATGTACGGAACAATGCGAGCCTCGTAGCGGAGGCCGCTTCCCGTGGTCACATCTCGTGCCTGACTACTAGTGGAC                                       >  1:284782/1‑301`  
                                                                                                                                                                                                                                                                         `ACCCTATGCTGACTCAGAGTAAAGAGGACTTGGTTGTATGGAAGGATTGATGACATTCGTAGTACGTACATCATTTCGTGAGGCCATTAAGGACATGCACGAAGAGTATGCAGATAAGGACTCTTTCAAACAATCTCCTGCAACAGTACGGGAGGTGTTCTGATGTCTGACTACCTGAAAGTGCTGCAAGCAATCAAAAGTTGCCCTAAGACTTTCCAGTCCAACTATGTACGGAACAATGCGAGCCTCGTAGCGGAGGCCGCTTCCCGTGGTCACATCTCGTGCCTGACTACTAGTGGAC                                       >  1:339272/1‑301`  
                                                                                                                                                                                                                                                                          `CCCTATGCTGACTCAGAGTAAAGAGGACTTGGTTGTATGGAAGGATTGATGACATTCGTAGTACGTACATCATTTCGTGAGGCCATTAAGGACATGCACGAAGAGTATGCAGATAAGGACTCTTTCAAACAATCTCCTGCAACAGTACGGGAGGTGTTCTGATGTCTGACTACCTGAAAGTGCTGCAAGCAATCAAAAGTTGCCCTAAGACTTTCCAGTCCAACTATGTACGGAACAATGCGAGCCTCGTAGCGGAGGCCGCTTCCCGTGGTCACATCTCGTGCCTGACTACTAGTGGACG                                      <  1:12469/301‑1`  
                                                                                                                                                                                                                                                                          `CCCTATGCTGAATCAGAGTAAAGAGGACTTGGTTGTATGGAAGGATTGATGACATTCGTAGTACGTACATCATTTCGTGAGGCCATTAAGGACATGCACGAAGAGTATGCAGATAAGGACTCTTTCAAACAATCTCCGGCAACAGTACGGGAGGTGTTCTGATGTCTGACTACCTGAAAGTGCTGCAAGCAATCAAAAGTTGCCCTAAGACTTTCCAGTCCAACTATGTACGGAACAATGCGAGCCTCGTAGCGGAGGCCGCTTCCCGTGGTCACATCTCGTGCCTGACTACTAGTGGACG                                      <  1:318570/301‑1`  
                                                                                                                                                                                                                                                                              `ATGCTGACTCAGAGTAAAGAGGACTTGGTTGTATGGAAGGATTGATGACATTCGTAGTACGTACATCATTTCGTGAGGCCATTAAGGACATGCACGAAGAGTATGCAGATAAGGACTCTTTCAAACAATCTCCTGCAACAGTACGGGAGGTGTTCTGATGTCTGACTACCTGAAAGTGCTGCAAGCAATCAAAAGTTGCCCTAAGACTTTCCAGTCCAACTATGTACGGAACAATGCGAGCCTCGTAGCGGAGGCCGCTTCCCGTGGTCACATCTCGTGCCTGACctgtctcttatacaca                                  >  1:236448/1‑285`  
                                                                                                                                                                                                                                                                                      `TCAGAGTAAAGAGGACTTGGTTGTATGGAAGGATTGATGACATTCGTAGTACGTACATCATTTCGTGAGGCCATTAAGGACATGCACGAAGAGTATGCAGATAAGGACTCTTTCAAACAATCTCCTGCAACAGTACGGGAGGTGTTCTGATGTCTGACTACCTGAAAGTGCTGCAAGCAATCAAAAGTTGCCCTAAGACTTTCCAGTCCAACTATGTACGGAACAATGCGAGCCTCGTATCGGAGGCCGCTTCCCGTGGTCACATCTCGTGCCTGACTACTAGTGGACGTAACGGTGGCGC                          <  1:300207/301‑1`  
                                                                                                                                                                                                                                                                                      `TCAGAGTAAAGAGGACTTGGTTGTATGGAAGGATTGATGACATTCGTAGTACGTACATCATTTCGTGAGGCCATTAAGGACATGCACGAAGAGTATGCAGATAAGGACTCTTTCAAACAATCTCCTGCAACAGTACGAGAGGTGTTCTGATGTCTGACTACCTGAAAGTGCTGCAAGCAATCAAAAGTTGCCCTAAGACTTTCCAGTCCAACTATGTACGGAACAATGCGAGCCTCGTAGCGGAGGCCGCTTCCCGTGGTCACATCTCGTGCCTGACTACTAGTGGACGTAACGGTGGCGC                          <  1:143410/301‑1`  
                                                                                                                                                                                                                                                                                             `AAAGAGGACTTGGTTGTATGGAAGGATTGATGACATTCGTAGTACGTACATCATTTCGTGAGGCCATTAAGGACATGCACGAAGAGTATGCAGATAAGGACTCTTTCAAACAATCTCCTGCAACAGTACGGGAGGTGTTCTGATGTCTGACTACCTGAAAGTGCTGCAAGCAATCAAAAGTTGCCCTAAGACTTTCCAGTCCAACTATGTACGGAGCAATGCGAGCCTCGTAGCGGAGGCCGCTTCCCGTGGTCACATCTCGTGCCTGACTACTAGTGGACGTAACGGTGGCGCTTGGGAA                   >  1:212450/1‑301`  
                                                                                                                                                                                                                                                                                             `AAAGAGGACTTGGTTGTATGGAAGGATTGATGACATTCGTAGTACGTACATCATTTCGTGAGGCCATTAAGGACATGCACGAAGAGTATGCAGATAAGGACTCTTTCAAACAATCTCCTGCAACAGTACGGGAGGTGTTCTGATGTCTGACTACCTGAAAGTGCTGCAAGCAATCAAAAGTTGCCCTAAGACTTTCCAGTCCAACTATGTACGGAACAATGCGAGCCTCGTAGCGGAGGCCGCTTCCCGTGGTCACATCTCGTGCCTGACTACTAGTGGACGTAACGGTGGCGCTTGGGAA                   >  1:72168/1‑301`  
                                                                                                                                                                                                                                                                                             `AAAGAGGACTTGGTTGTATGGAAGGATTGATGACATTCGTAGTACGTACATCATTTCGTGAGGCCATTAAGGACATGCACGAAGAGTATGCAGATAAGGACTCTTTCAAACAATCTCCTGCAACAGTACGGGAGGTGTTCTGATGTCTGACTACCTGAAAGTGCTGCAAGCAATCAAAAGTTGCCCTAAGACTTTCCAGTCCAACTATGTACGGAACAATGCGAGCCTCGTAGCGGAGGCCGCTTCCCGTGGTCACATCTCGTGCCTGACTACTAGTGGACGTAACGGTGGCGCTTGGGAA                   >  1:254414/1‑301`  
                                                                                                                                                                                                                                                                                             `AAAGAGGACTTGGATGTATGGAAGGATTGATGACATTCGTAGTACGTACATCATTTCGTGAGGCCATTAAGGAAATGCACGAAGAGTATGCAGATAAGGACTCTTTCCAACAATCTCCTGCAACAGTACGGGATGTGTTCTGATGTCTGACTACCTGACAGTGCTGCAAGCAATCAAAAGTTGCCCTAAGACTTTCCAGTCCAACTATGTACGGAACAATGCGAGCCTCGTAGCGGAAGCCGCTTCCCGTGTTCACATCTCACGCCTGACTACTAGTGGACGTAACGGTGACGCTTGGGAA                   >  1:28202/1‑301`  
                                                                                                                                                                                                                                                                                                   `GACTTGGTTGTATGGAAGGATTGATGACATTCGTAGTACGTACATCATTTCGTGAGGCCATTAAGGACATGCACGAAGAGTATGCAGATAAGGACTCTTTCAAACAATCTCCTGCAACAGTACGGGAGGTGTTCTGATGTCTGACTACCTGAAAGTGCTGCAAGCAATCAAAAGTTGCCCTAAGACTTTCCAGTCCAACTATGTACGGAACAATGCGAGCCTCGTAGCGGAGGCCGCTTCCCGTGGTCACATCTCGTGCCTGACTACTAGTGGACGTAACGGTGGCGCTTGGGAAATCACT             >  1:1693/1‑301`  
                                                                                                                                                                                                                                                                                                    `ACTTGGTTGTATGGAAGGATTGATGACATTCGTAGTACGTACATCATTTCGTGAGGCCATTAAGGACATGCACGAAGAGTATGCAGATAAGGACTCTTTCAAACAATCTCCTGCAACAGTACGGGAGGTGTTCTGATGTCTGACTACCTGAAAGTGCTGCAAGCAATCAAAAGTTGCCCTAAGACTTTCCAGTCCAACTATGTACGGAACAATGCGAGCCTCGTAGCGGAGGCCGCTTCCCGTGGTCACATCTCGTGCCTGACTACTAGTGGACGTAACGGTGGCGCTTGGGAAATCACTG            >  1:233666/1‑301`  
                                                                                                                                                                                                                                                                                                            `GTATGGAAGGATTGATGACATTCGTAGTACGTACATCATTTCGTGAGGCCATTAAGGACATGCACGAAGAGTATGCAGATAAGGACTCTTTCAAACAATCTCCTGCAACAGTACGGGAGGTGTTCTGATGTCTGACTACCTGAAAGTGCTGCAAGCAATCAAAAGTTGCTCTAAGACCTTCCAGTCCAACTATGTACGGAACAATGCGAGCCTCGTAGCGGAGGCCGCTTCCCGTGGTCACATCTCGTGCCTGACTACTAGTGGACGTAACGGTGGCGCTTGGGAAATGACTGCTTCCGGT    >  1:17405/1‑301`  
                                                                                                                                                                                                                                                                                                            `GTATGGAAGGATTGATGACATTCGTAGTACGTACATCATTTCGTGAGGCCATTAAGGACATGCACGAAGAGTATGCAGATAAGGACTCTTTCAAACAATCTCCTGCAACAGTACGGGAGGTGTTCTGATGTCTGACTACCTGAAAGTGCTGCAAGCAATCAAAAGTTGCCCTAAGACTTTCCAGTCCAACTATGTACGGAACAATGCGAGCCTCGTATCGGAGGCCGCTTCCCGTGGTCACATCTCGTGCCTGACTACTAGTGGACGTAACGGTGGCGCTTGGGAAATCACTGCGTCTGGT    >  1:222662/1‑301`  
                                                                                                                                                                                                                                                                                                            `GTATGGAAGGATTGATGACATTCGTAGTACGTACATCATTTCGTGAGGCCATTAAGGACATGCACGAAGAGTATGCAGATAAGGACTCTTTCAAACAATCTCCTGCAACAGTACGGGAGGTGTTCTGATGTCTGACTACCTGAAAGTGCTGCAAGCAATCAAAAGTTGCCCTAAGACTTTCCAGTCCAACTATGTACGGAACAATGCGAGCCTCGTAGCGGAGGCCGCTTCCCGTGGTCACATCTCGTGCCTGACTACTAGTGGACGTAACGGTGGCGCTTGGGAAATCACTGCTTCCGGT    >  1:200051/1‑301`  
                                                                                                                                                                                                                                                                                                            `GTATGGAAGGATTGATGACATTCGTAGTACGTACATCATTTCGTGAGGCCATTAAGGACATGCACGAAGAGTATGCAGATAAGGACTCTTTCAAACAATCTCCTGCAACAGTACGGGAGGTGTTCTGATGTCTGACTACCTGAAAGTGCTGCAAGCAATCAAAAGTTGCCCTAAGACTTTCCAGTCCAACTATGTACGGAACAATGCGAGCCTCGTAGCGGAGGCCGCTTCCCGTGGTCACATCTCGTGCCTGACTACTAGTGGACGTAACGGTGGCGCTTGGGAAATCACTCCTTCCGGT    >  1:262967/1‑301`  
                                                                                                                                                                                                                                                                                                            `GTATGGAAGGATTGATGACATTCGTAGTACGTACATCATTTCGTGAGGCCATTAAGGACATGCACGAAGAGTATGCAGATAAGGACTCTTTCAAACAATCTCCTGCAACAGTACGGGAGGTGTTCTGATGTCTGACTACCTGAAAGTGCTGCAAGCAATCAAAAGTTGCCCTAAGACTTTCCAGTCCAACTATGTACGGAACAATGCGAGCCTCGTAGCGGAGGCCGCTTCCCGCGGTCACATCTCGTGCCTGACTACTAGTGGACGTAACGGTGGCGCTTGGGAAATCACTGCTTCCGat    >  1:181258/1‑299`  
                                                                                                                                                                                                                                                                                                              `ATGGAAGGATTGATGACATTCGTAGTACGTACATCATTTCGTGAGGCCATTAAGGACATGCACGAAGAGTATGCAGATAAGGACTCTTTCAAACAATCTCCTGCAACAGTACGGGAGGTGTTCTGATGTCTGACTACCTGAAAGTGCTGCAAGCAATCAAAAGTTGCCCTAAGACTTTCCAGTCCAACTATGTACGGAACAATGCGAGCCTCGTAGCGGAGGCCGCTTCCCGTGGTCACATCTCGTGCCTGACTACTAGTGGACGTAACGGTGGCGCTTGGGAAATCACTGCTTCCGGTAC  >  1:204775/1‑301`  

                                                                                                                                                                                                                                                                                                                                                                                                                                                                                                                                                                                                                             
`GCATGGGTACATGATGAAATCCAAGTAGGCTGCCGTACCGAAGAGATTGCTCAGGTGGTCATTGAGACCGCACAAGAAGCGATGCGCTGGGTTGGAGACCACTGGAACTTCCGGTGTCTTCTGGATACCGAAGGTAAGATGGGTCCTAATTGGGCGATTTGCCACTGATACAGGAGGCTACTCATGAACGAAAGACACTTAACAGGTGCTGCTTCTGAAATGCTAGTAGCCTACAAATTTACCAAAGCTGGGTACACTGTCTATTACCCTATGCTGACTCAGAGTAAAGAGGACTTGGTTGTATG‑‑‑‑‑‑‑‑‑‑‑‑‑‑‑‑‑‑‑‑‑‑‑‑‑‑‑‑‑‑‑‑‑‑‑‑‑‑‑‑‑‑‑‑‑‑‑‑‑‑‑‑‑‑‑‑‑‑‑‑‑‑‑‑‑‑‑‑‑‑‑‑‑‑‑‑‑‑‑‑‑‑‑‑‑‑‑‑‑‑‑‑‑‑‑‑‑‑‑‑‑‑‑‑‑‑‑‑‑‑‑‑‑‑‑‑‑‑‑‑‑‑‑‑‑‑‑‑‑‑‑‑‑‑‑‑‑‑‑‑‑‑‑‑‑‑‑‑‑‑‑‑‑‑‑‑‑‑‑‑‑‑‑‑‑‑‑‑‑‑‑‑‑‑‑‑‑‑‑‑‑‑‑‑‑‑‑‑‑‑‑‑‑‑‑‑‑‑‑‑‑‑‑‑‑‑‑‑‑‑‑‑‑‑‑‑‑‑‑‑‑‑‑‑‑‑‑‑‑‑‑‑‑‑‑‑‑‑‑‑‑‑‑‑‑‑‑‑‑‑‑‑‑‑‑‑‑‑‑‑‑‑‑‑‑‑‑‑‑‑‑‑‑‑‑‑‑‑‑‑‑‑‑‑‑‑‑‑‑‑‑‑‑‑‑‑‑‑  >  NC_001604.1/16300‑16604`  
`‑‑‑‑‑‑‑‑‑‑‑‑‑‑‑‑‑‑‑‑‑‑‑‑‑‑‑‑‑‑‑‑‑‑‑‑‑‑‑‑‑‑‑‑‑‑‑‑‑‑‑‑‑‑‑‑‑‑‑‑‑‑‑‑‑‑‑‑‑‑‑‑‑‑‑‑‑‑‑‑‑‑‑‑‑‑‑‑‑‑‑‑‑‑‑‑‑‑‑‑‑‑‑‑‑‑‑‑‑‑‑‑‑‑‑‑‑‑‑‑‑‑‑‑‑‑‑‑‑‑‑‑‑‑‑‑‑‑‑‑‑‑‑‑‑‑‑‑‑‑‑‑‑‑‑‑‑‑‑‑‑‑‑‑‑‑‑‑‑‑‑‑‑‑‑‑‑‑‑‑‑‑‑‑‑‑‑‑‑‑‑‑‑‑‑‑‑‑‑‑‑‑‑‑‑‑‑‑‑‑‑‑‑‑‑‑‑‑‑‑‑‑‑‑‑‑‑‑‑‑‑‑‑‑‑‑‑‑‑‑‑‑‑‑‑‑‑‑‑‑‑‑‑‑‑‑‑‑‑‑‑‑‑‑‑‑‑‑‑‑‑‑‑‑‑‑‑‑‑‑‑‑‑‑‑‑‑‑‑‑‑‑‑‑‑‑‑‑‑‑gtatgGAAGGATTGATGACATTCGTAGTACGTACATCATTTCGTGAGGCCATTAAGGACATGCACGAAGAGTATGCAGATAAGGACTCTTTCAAACAATCTCCTGCAACAGTACGGGAGGTGTTCTGATGTCTGACTACCTGAAAGTGCTGCAAGCAATCAAAAGTTGCCCTAAGACTTTCCAGTCCAACTATGTACGGAACAATGCGAGCCTCGTAGCGGAGGCCGCTTCCCGTGGTCACATCTCGTGCCTGACTACTAGTGGACGTAACGGTGGCGCTTGGGAAATCACTGCTTCCGGTAC  >  NC_001604.1/17028‑17325`

  

Alignment Legend

Aligned base mismatch/match (shaded by quality score): `ATCG/ATCG` `< 3 ≤` `ATCG/ATCG` `< 11 ≤` `ATCG/ATCG` `< 24 ≤` `ATCG/ATCG` `< 35 ≤` `ATCG/ATCG` `< 38 ≤` `ATCG/ATCG`

Unaligned base: `atcg`    Masked matching base: `atcg`    Alignment gap: `‑`    Deleted base: `‑`

Reads not counted as support for junction

read\_name Not counted due to insufficient overlap past the breakpoint.

read\_name Not counted due to not crossing MOB target site duplication.
